# Hospital Surgical Volume Is Poorly Correlated With Delivery of Multimodal Treatment for Localized Pancreatic Cancer: A National Retrospective Cohort Study

**DOI:** 10.1097/AS9.0000000000000197

**Published:** 2022-08-17

**Authors:** Benjamin D. Powers, James McDonald, Rahul Mhaskar, Simon J. Craddock Lee, Jennifer B. Permuth, Susan Vadaparampil, Scott M. Gilbert, Jason W. Denbo, Dae Won Kim, Jose M. Pimiento, Pamela J. Hodul, Mokenge P. Malafa, Daniel A. Anaya, Jason B. Fleming

**Affiliations:** From the * Department of Gastrointestinal Oncology, Moffitt Cancer Center, Tampa, FL; † Department of Health Outcomes and Behavior, Moffitt Cancer Center, Tampa, FL; ‡ University of South Florida Morsani College of Medicine, Tampa, FL; § University of Texas Southwestern Simmons Comprehensive Cancer Center, Dallas, TX; ∥ Department of Cancer Epidemiology, Moffitt Cancer Center, Tampa, FL; ¶ Department of Urologic Oncology, Moffitt Cancer Center, Tampa, FL.

**Keywords:** care coordination, multimodal treatment, pancreatic cancer, surgical volume

## Abstract

Using Donabedian’s quality of care model, this study assessed process (hospital multimodal treatment) and structure (hospital surgical case volume) measures to evaluate localized pancreatic cancer outcomes.

**Background:**

Treatment at high surgical volume hospitals has been shown to improve short-term outcomes. However, multimodal treatment—surgery and chemotherapy—is the standard of care yet only received by 35% of US patients and has not been examined at the hospital level.

**Methods:**

The National Cancer Database was used to identify a cohort of clinical stage I pancreatic cancer patients eligible for multimodal treatment from 2004 to 2016. Hospital multimodal treatment was defined as the number of patients receiving surgery and chemotherapy by the number of eligible patients per hospital. Descriptive statistics and survival analyses were conducted.

**Results:**

A total of 16,771 patients met inclusion criteria, of whom 68.0% received curative-intent surgery and 35.8% received multimodal treatment. There was poor correlation between hospital surgical volume and delivery of multimodal treatment (Spearman correlation 0.214; *P* < 0.001). Of patients cared for at the highest surgical volume hospitals, 18.8% and 52.1% were treated at hospitals with low (0%–25%) and moderate (>25%–50%) multimodal treatment delivery, respectively. Higher hospital multimodal treatment delivery was associated with improved overall survival.

**Discussion:**

Although the volume–outcome relationship for pancreatic cancer has demonstrated improved outcomes, this work identified poor correlation between hospital surgical volume and delivery of multimodal treatment. The role of care coordination in the delivery of multimodal treatment warrants further investigation as it is associated with improved survival for patients with localized pancreatic cancer.

## INTRODUCTION

Since the early 1980s, the volume–outcome relationship has held a prominent position in health policy discourse and become an overriding goal to produce high-quality cancer outcomes.^1^ As data showing the impact of surgical volume on outcomes grew, the Institute of Medicine (now National Academy of Medicine) recommended that cancer patients undergoing technically complex procedures be treated at high-volume centers.^1,2^ Studies continue to show the benefit of high-volume care and payers and patient-advocacy groups have worked to steer patients to high-volume hospitals for procedures such as pancreatoduodenectomy.^3,4^ Highlighting its importance, a recent review stated that the volume–outcome relationship “has revolutionized delivery of complex cancer care with resultant large-scale improvements in patient outcomes.”^5^

The volume–outcome relationship is associated with the Donabedian model of healthcare quality, which describes the impact of structure, process, and outcomes on quality of care (Supplemental File 1, http://links.lww.com/AOSO/A160).^6^ Structure refers to “the settings in which care occurs,” and includes material resources (eg, health facilities) as well as human resources (eg, the number and qualifications of personnel).^7^ Process describes “what is actually done in giving and receiving care” and refers to the activities that constitute health care: patients seeking and receiving a care plan as well as practitioners’ role in evaluating, recommending, and delivering treatment.^7^ Within this model, hospital volume is a structural factor, dependent on the setting of care. Consequently, the volume–outcome relationship model does not explicitly include processes of care, such as the coordination and delivery of complex, multidisciplinary treatment.

For many localized gastrointestinal cancers, multidisciplinary-guided multimodal treatment is the standard of care.^8–10^ Taking localized pancreatic ductal adenocarcinoma (hereafter, pancreatic cancer) as the archetype, early-stage disease has increasingly been viewed as a systemic disease. For nearly 2 decades, the best outcomes have been achieved through multidisciplinary, multimodal treatment.^11,12^ While optimal sequences and regimens remain unclear, there is consensus that multimodal treatment offers improved outcomes over surgery alone.^13–15^ An underlying assumption of the volume–outcome relationship is that higher surgical volume treatment facilities are also high-performing at process measures such as care coordination and the multidisciplinary delivery of treatment. However, few studies have evaluated the role of process measures, such as the coordination and delivery of multimodal treatment for pancreatic cancer, at the hospital level.^16^

To address this knowledge gap, this disparities study investigated the delivery of multimodal treatment as a process measure at health care facilities using a national database. Process, as measured by hospital multimodal treatment, and structure, measured by hospital surgical case volume, were used to assess pancreatic cancer outcomes. We hypothesized that there would be variation in the delivery of multimodal treatment by hospital surgical volume and this hospital-based process measure of care coordination would be associated with overall survival (OS).

## METHODS

### Data Source and Study Population

This study was a retrospective cohort exploratory analysis that used the National Cancer Database (NCDB) participant user file from 2004 to 2016. The NCDB is a nationwide, facility-based surveillance resource that collects hospital registry data from nearly 1500 Commission on Cancer–accredited facilities and comprises approximately 70% of newly diagnosed cancer cases in the United States.^17^ It is sponsored by the Commission on Cancer of the American College of Surgeons and the American Cancer Society. Because the data were de-identified, this study was exempt from Institutional Review Board review.

A cohort of patients with localized (clinical stage I per AJCC 8th edition criteria) pancreatic cancer was identified (Supplemental File 2, http://links.lww.com/AOSO/A160). Patients receiving neoadjuvant treatment were excluded due to potential confounding of disease progression, which introduce bias into the study. Given the limitations of the dataset to identify whether patients did not receive surgery due to tumor biology or because of process factors like care coordination, the cohort was limited to upfront surgery patients. Pancreatic cancer was defined by codes 8140, 8480, 8481, and 8500 based on the International Classification of Disease for Oncology, 3rd Edition. Curative-intent surgery was defined based on site-specific surgery of the primary site using codes 30–80. Patients with unknown data for surgery, chemotherapy, or clinical stage were excluded. Patients receiving all treatment at a nonreporting facility were excluded.

### Variables

**FIGURE 1.**
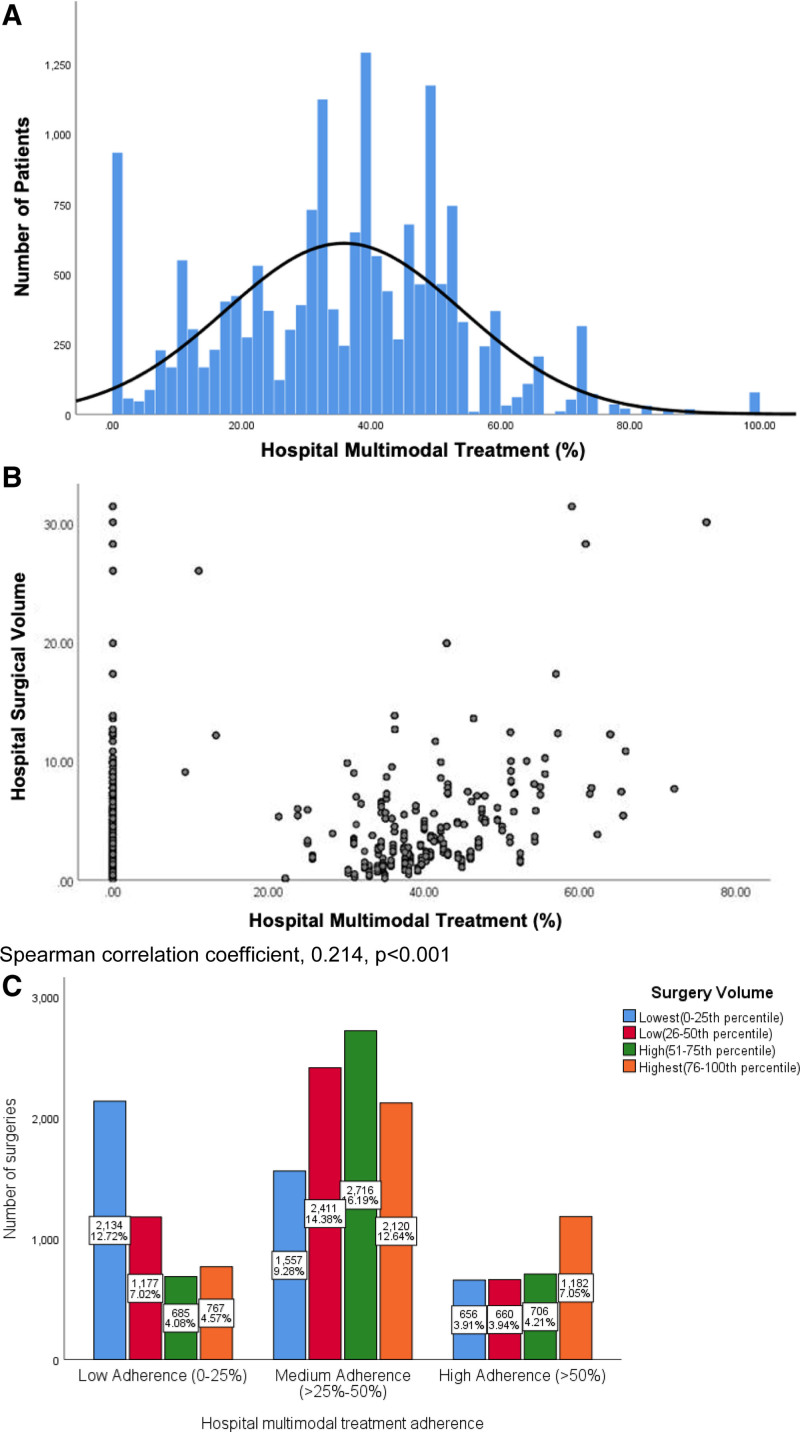
Characteristics of hospital multimodal treatment. A, Distribution of hospital delivery of multimodal treatment. B, Scatterplot of hospital surgical volume and hospital multimodal treatment rate. C, Hospital delivery of multimodal treatment by hospital surgical volume.

The predictor variable was the proportion of multimodal treatment delivery at the facility level. Multimodal treatment was defined as receipt of curative-intent surgery and adjuvant chemotherapy. Hospital proportion of multimodal treatment delivery was determined by the number of patients receiving multimodal treatment over the total number of patients receiving care at that hospital. To identify points of categorization for this continuous variable, the frequency distribution was graphically evaluated (Fig. 1) and hospital multimodal treatment was categorized as low (0%–25%), moderate (>25%–50%), and high (>50%). The outcome variable was OS and was defined as the time from diagnosis to death or last follow-up. Covariates included age, sex, race, ethnicity, insurance status, Charlson-Deyo comorbidity index, facility type, analytic stage, surgery type, tumor grade, and facility surgical volume. Facility surgical volume was categorized into quartiles and was designated before excluding clinical stage II patients and patients who underwent a neoadjuvant approach to ensure an accurate representation of pancreatic surgery volume, regardless of treatment sequence.

### Statistical Analysis

Patient characteristics were summarized using descriptive statistics, including frequencies and proportions for categorical measures and median and 25th and 75th percentiles for continuous measures. The association between hospital multimodal treatment and demographic and clinical factors was investigated via χ^2^ or Fisher exact test and the Kruskal Wallis test. The relationship between hospital surgical volume and hospital multimodal treatment was assessed with the nonparametric Spearman correlation. The Kaplan-Meier analysis was conducted to estimate the median OS time and 95% confidence intervals for hospital multimodal treatment adherence categories. The differences in the OS times of the hospital multimodal treatment adherence categories were investigated by the log-rank test. The predictors of OS time were investigated by the univariate and multivariate Cox proportional hazards regression. *P* values less than 0.05 indicated statistical significance. Analyses were conducted using SPSS version 26 and MedCalc version 20.011 software.

## RESULTS

### Characteristics of the Study Population

Characteristics of the 16,771 patients who met inclusion criteria are shown in Table 1 by hospital multimodal treatment. Curative-intent surgery was delivered to 68.0% and multimodal treatment was delivered to 35.8% of patients. Figure 1A demonstrates the distribution of hospital multimodal treatment.

**TABLE 1.** Baseline Characteristics of Resectable Pancreatic Cancer by Hospital Multimodal Treatment Delivery (n = 16,771)

	Hospital Multimodal Treatment Delivery			*P*
	Low (0%–25%)	Moderate (>25%–50%)	High (>50%)	
Total	4763 (28.4)	8804 (52.5)	3204 (19.1)	
Age	73 (27.0,90.0)	70 (28.0,90.0)	69 (26.0,90.0)	***<0.001***
Sex				0.226
Female	2535 (53.2)	4636 (52.7)	1643 (51.3)	
Male	2228 (46.8)	4168 (47.3)	1561 (48.7)	
Race				***<0.001***
White	3908 (82.0)	7487 (85.0)	2799 (87.4)	
Black	623 (13.1)	936 (10.6)	280 (8.7)	
Other/unknown	232 (4.9)	381 (4.3)	125 (3.9)	
Ethnicity				***<0.001***
Hispanic	290 (6.1)	217 (2.5)	102 (3.2)	
Non-Hispanic	4296 (90.2)	8166 (92.8)	3017(94.2)	
Unknown	177 (3.7)	421 (4.8)	85 (2.7)	
Insurance				***<0.001***
Private	1247 (26.6)	2601 (29.9)	1023 (32.2)	
Medicare	3010 (64.2)	5464 (62.7)	1976 (62.1)	
Medicaid	220 (4.7)	381 (4.4)	100 (3.1)	
Uninsured	139 (3.0)	146 (1.7)	52 (1.6)	
Other government	70 (1.5)	117 (1.3)	31 (1.0)	
Facility type				***<0.001***
Academic	1708 (35.9)	5126 (58.2)	1567 (48.9)	
Comprehensive Community Cancer Center	2115 (44.4)	2261 (25.7)	914 (28.5)	
Integrated Network Cancer Center	553 (11.6)	1131(12.8)	592 (18.5)	
Community Cancer Center	367 (7.7)	250 (2.8)	108 (3.4)	
Unknown	20 (0.4)	36 (0.4)	23 (0.7)	
Surgery type				***<0.001***
None	3013 (63.3)	2772 (31.5)	480 (15.0)	
Whipple	1164 (24.4)	4135 (47.0)	1902 (59.4)	
Distal pancreatectomy	374 (7.9)	1129 (12.8)	527 (16.4)	
Total pancreatectomy	212 (4.5)	768 (8.7)	295 (9.2)	
Hospital surgery volume				***<0.001***
Lowest (0%–25%)	2134 (44.8)	1557 (17.7)	656 (20.5)	
Low (>25%–50%)	1177 (24.7)	2411 (27.4)	660 (20.6)	
High (>50%–75%)	685 (14.4)	2716 (30.9)	706 (22.0)	
Highest (>75%)	767 (16.1)	2120 (24.1)	1182 (36.9)	
Patient multimodal treatment				***<0.001***
No treatment	1963 (41.2)	1559 (17.7)	271 (8.5)	
No multimodal treatment	2181 (45.8)	3795 (43.1)	1001 (31.2)	
Multimodal treatment	619 (13.0)	3450 (39.2)	1932 (60.3)	

Bold values indicate statistical significance at less than or equal to 0.05

More than 60% of patients treated with multimodal therapy received their care at high multimodal propensity hospitals. However, for moderate and low multimodal treatment hospitals, multimodal treatment was delivered to 39.2% and 13.0% of patients, respectively. Low multimodal treatment hospitals delivered no treatment to 41.2% of patients. Moderate and high multimodal treatment hospitals delivered no treatment to 17.7% and 8.5% of patients, respectively.

There were differences in hospital multimodal treatment and demographic and clinical factors. A higher proportion of patients who were older, Black, Hispanic, or had public insurance (ie, Medicare or Medicaid) or uninsured insurance status were treated at hospitals with low multimodal treatment delivery. High multimodal delivery hospitals treated a higher proportion of White and privately insured patients. A higher proportion of patients with no Charlson comorbidities were treated at low multimodal delivery hospitals; however, patients with higher Charlson comorbidity scores tended to be treated at high multimodal delivery hospitals. Additionally, there were differences by facility type. Of patients cared for at academic facilities, 20.3% received care at a low multimodal delivery hospital. Of patients cared for at comprehensive community cancer centers, 40.0% received care at a low multimodal delivery hospital.

There were also differences in hospital multimodal treatment delivery by hospital surgical volume (Fig. 1C). Of patients treated at the lowest surgical volume hospitals, 44.8% of patients were cared for at a hospital with low multimodal treatment delivery. Of patients cared for at the highest surgical volume hospitals, 36.9% of patients were treated at a hospital with high multimodal treatment delivery. However, 18.8% of patients cared for at the highest surgical volume hospitals received care at a hospital with low multimodal delivery. Conversely, 15.1% of patients received care at a hospital with the lowest surgical volume but high multimodal treatment delivery. Figure 1B depicts a scatterplot of hospital surgical volume and hospital multimodal treatment rate. The Spearman correlation coefficient was 0.214, *P* < 0.001, demonstrating poor correlation between hospital surgical volume and hospital multimodal treatment delivery.

### Overall Survival

**FIGURE 2.**
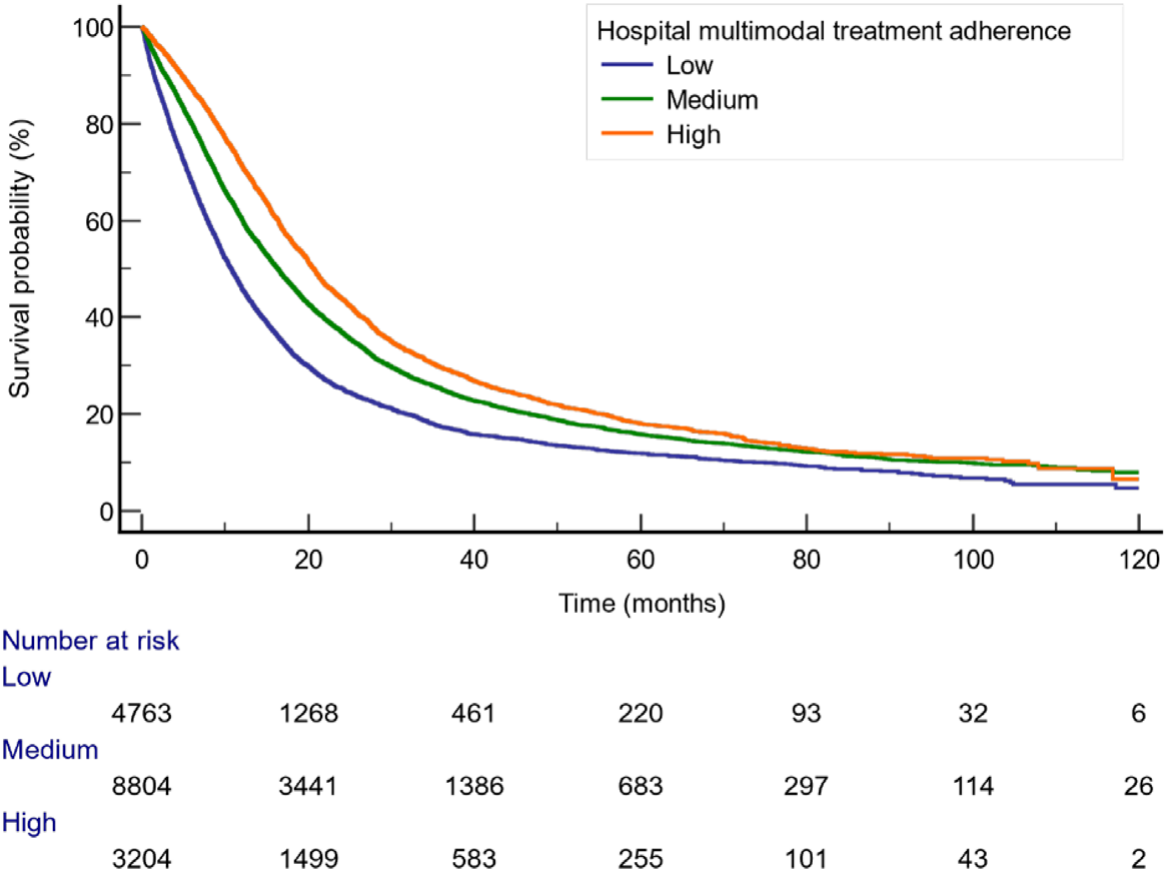
Kaplan-Meier curves of the probability of overall survival by hospital receipt of multimodal treatment.

The median OS was 15.4 months [95% confidence interval (CI), 15.1–15.8]. There were differences in OS by hospital multimodal treatment (Fig. 2). The median OS of the lowest hospital surgical volume quartile was 12.0 months (95% CI, 11.4–12.5). Median OS of the highest hospital surgical volume quartile was 19.2 months (95% CI, 18.4–20.0). Table 2 shows that the median OS for patients treated at a hospital with a low multimodal treatment was 10.8 months (95% CI, 10.2–11.2. The median OS was 20.7 months (95% CI, 19.8–21.6) for those treated at a high multimodal treatment hospital. In regression analysis, hospital multimodal treatment was associated with OS (Table 3). Adjusted analysis showed that receiving care at a hospital with moderate or high multimodal treatment deliver was associated with a lower hazard of death relative to treatment at a low multimodal hospital (hazard ratio 0.91; 95% CI, 0.87–0.95 and hazard ratio 0.81; 95% CI, 0.77–0.86, respectively).

**TABLE 2.** Absolute Overall Survival Estimates

	N	Events	Median (95% CI)	*P*
Overall	16,771	13,209	15.4 (15.1–15.8)	
Hospital multimodal treatment				***<0.001***
Low (0%–25%)	4763	3917	10.8 (10.2–11.2)	
Moderate (>25%–50%)	8804	6904	16.4 (15.9–16.9)	
High (>50%)	3204	2388	20.7 (19.8–21.6)	
Hospital surgical volume				***<0.001***
Lowest (0%–25%)	4347	3604	12.0 (11.4–12.5)	
Low (>25%–50%)	4248	3418	14.0 (13.3–14.6)	
High (>50%–75%)	4107	3096	17.5 (16.8–18.3)	
Highest (>75%)	4069	3091	19.2 (18.4–20.0)	

Bold values indicate statistical significance at less than or equal to 0.05.

**TABLE 3.** Relative Hazard of Death: Univariate and Multivariable Cox Proportional Regression Model

	Univariate		Multivariable*	
	HR (95% CI)	*P*	HR (95% CI)	*P*
Hospital multimodal treatment (ref: low)		***<0.001***		***<0.001***
Moderate	0.73 (0.70–0.76)		0.91 (0.87–0.95)	
High	0.61 (0.58–0.64)		0.81 (0.77–0.86)	

Bold values indicate statistical significance at less than or equal to 0.05.

*Multivariable model adjusted for age, Charlson comorbidity score, race, insurance status, grade, stage, and hospital surgical volume.

## DISCUSSION

In this study a process measure, hospital delivery of multimodal therapy, was associated with OS in localized pancreatic cancer. Furthermore, there was poor correlation between hospital surgical volume and delivery of multimodal treatment; nearly 20% of patients were cared for at high surgical volume facilities that delivered no or a low rate of multimodal treatment. These results suggest that policies solely employing the volume-outcome relationship are incomplete reflections of localized pancreatic cancer treatment quality and potentially encourage treatment at high-volume facilities with suboptimal care coordination and delivery of multimodal treatment.

Prior studies have demonstrated that surgery at high-volume centers for pancreatic cancer is associated with improved outcomes. With the publication of several articles at the turn of the 21st century, the volume–outcome relationship burgeoned and became a research and policy strategy to improve treatment quality.^18–20^ Responses within the surgical field have ranged from advocating for a “Take the Volume Pledge” campaign to place limits on surgical procedures at low-volume centers to calling for the complete elimination of surgery at low-volume centers; others addressed the ethics of sending cancer patients to low-volume hospitals.^21–23^ The Leapfrog Group, a nonprofit organization that consists of public and private health care purchasers and is self-described as “America’s voice for patient safety,” publishes minimum hospital volume standards for pancreatic resection for cancer to encourage patients to seek higher-volume hospitals for surgery.^3^ Such recommendations have been embraced by patient-advocacy groups such as the Pancreatic Cancer Action Network, which strongly recommends patients seek high-volume care.^4^

Historically, surgery has been the primary treatment for localized pancreatic cancer and quality improvement efforts that focused on surgical outcomes had great resonance. However, the publication of the European study group for pancreatic cancer-1 trial in 2004 recommended adjuvant chemotherapy as the new standard of care for pancreatic cancer, and the final results of the Charité Onkologie 001 trial in 2008, which showed adjuvant chemotherapy for pancreatic cancer provided superior OS, offered level-one evidence for the multimodal treatment of localized pancreatic cancer.^24,25^ This approach became the standard of care, with clinical trial data of multimodal treatment demonstrating a median OS of 54.4 months.^12^ While the exact sequence of treatment remains uncertain, multimodal treatment has been embraced by national and organizational guidelines, with some authors now describing localized pancreatic cancer as a systemic disease that requires systemic treatment.^11,26^

Although multidisciplinary, multimodal treatment has become the standard of care for several early-stage gastrointestinal cancers, the explicit study of process measures for cancer treatment delivery has generally failed to garner significant research attention (with the exception of chronic conditions and survivorship).^27,28^ The potential consequences of poor care coordination are significant—national data suggest that about half of patients with resectable pancreatic cancer and who are healthy enough to undergo surgery do not receive it; furthermore, only 35% receive multimodal treatment.^29–33^ Described as a “system in crisis,” the National Academy of Medicine has reported that cancer patients’ care is often poorly coordinated, which leads to less treatment and higher costs.^34^ The process of cancer care coordination, defined as the multilevel, deliberate organization of patient care between stakeholders (the patient, caregivers, providers, and organizations), remains a complex and understudied problem.^35^ While some studies have introduced the importance of broader process measures and attempted to incorporate them into volume–outcome research, few have investigated the multilevel (patient, provider, and health system) role of care coordination in multimodal cancer treatment.^16,36^ Vissers et al^37^ evaluated failure to comply with National Comprehensive Cancer Network guidelines for pancreatic cancer and found that guideline-concordant treatment ranged from 5% to 57% among 50 large hospitals in California, highlighting the significant treatment delivery variation between institutions. Nevertheless, the inputs and best practices required to improve care coordination for optimal delivery of multimodal treatment for cancer patients are unknown.

Although Donabedian described the importance and iterative nature of process measures across a patients’ course of care (from diagnosis to testing to treatment and follow-up), conceptualization of process measures within surgical research discourse have frequently been limited to the perioperative surgical episode.^6^ Here, process measures have focused on antibiotic administration, Foley catheter removal, and venous thromboembolism prophylaxis.^38,39^ Instead of conceiving of treatment quality as coordinated actions across the (cancer) care continuum, processes of care have been superseded by structural measures based on the assumption that a “shared system” may account for improved outcomes.^40^ This single-episode view of treatment quality has likely played a role in the description of process measures as a “proxy” of surgical volume.^41,42^ This may in part explain the evolution of the structure-process-outcome model to the volume–outcome relationship and suggest why contemporary studies assessing cancer treatment disparities have rarely acknowledged process measures, like closing the referral loop and addressing care coordination. Instead, volume–outcome studies have typically concluded that marginalized and under-resourced populations need care from high-volume surgeons/facilities but failed to offer a blueprint for how to achieve this.^43,44^ The data presented in this study view racial disparities as a process measure: the highest proportion of Black and Hispanic patients were treated at low multimodal treatment hospitals. Identifying the actions and mechanisms by which the referral process and care coordination function could offer a path to create interventions to reduce racial and ethnic cancer treatment disparities. This direction also breaks the impasse of policy recommendations targeting only the volume–outcome relationship, which, as a quality of care framework, does not address the “activities of health care” and is unequipped to address barriers such as racism, poverty, or, more broadly, structural violence, that preclude or disrupt closure of the referral loop, care coordination, and ultimately, multimodal treatment.^45,46^

Despite the lack of focus on process measures for resectable gastrointestinal cancers, there has been increasing appreciation of the role of care coordination in incentive-based models, such as the Centers for Medicare & Medicaid Services’ implementation of the Merit-based Incentive Payment System. Merit-based Incentive Payment System has targeted care coordination measures as an improvement area for physicians and quality requirements, which contribute the greatest weight to reimbursement, include 34 process/care coordination measures.^47^ One such measure, closing the specialist referral loop, is defined as the percentage of patients who complete a referral and the referring provider receives a report of that encounter.^48^ With reimbursement adjustments reaching 9% in 2022 and data suggesting that high social-risk patients may negatively impact reimbursement, there is also a financial incentive to better define and investigate the role of care coordination in complex cancer treatment delivery.^49,50^

There are several limitations to consider when interpreting findings from this study, including those related to retrospective analysis and the use of a large national database. Data for NCDB cases are abstracted by certified registrars who include all available medical records, including cancer care that is performed outside of the reporting facility, such as adjuvant systemic treatment. Although steps are taken to ensure comprehensive data collection, records may be incomplete, which could impact these results. The NCDB is does not collect complication data and the impact cannot be analyzed. Furthermore, pre-operative evaluation (staging), follow-up, and recurrence data are not collected. Another limitation is the ability to identify treatment quality considering more facilities are employing a neoadjuvant strategy for all pancreatic cancer patients. Given the variables in the NCDB, it is unclear how to determine which patients failed to undergo surgery after neoadjuvant treatment due to process barriers from those who had progression of disease and were therefore no longer a candidate for surgery. While this makes interpreting treatment quality for patients undergoing a neoadjuvant approach challenging, the generalizability of these results also suffers by focusing only on a surgery-first cohort. This will likely remain a challenge for future studies of treatment quality for resectable gastrointestinal malignancies using national datasets. Finally, granular measures of socioeconomic status are not available and can not be excluded as a potential confounder.

In summary, these data demonstrate that a process measure, the coordination of multimodal treatment delivery, is associated with OS for resectable pancreatic cancer. Furthermore, the correlation between hospital surgical volume and delivery of multimodal treatment is poor. As multimodal treatment has become the standard of care for resectable pancreatic cancer, more research is needed to investigate the role of process measures of treatment quality, such as care coordination, to improve outcomes for all patients.

## ACKNOWLEDGMENTS

I acknowledge and confirm that all co-authors have seen and agreed to the contents of the manuscript. Each author participated extensively in this original work that he/she may take public responsibility for respective portions of the content and all meet the conditions outlined for authorship. B.D.P. contributed to study conceptualization/design. B.D.P., R.M. contributed to data curation. R.M. contributed to formal analysis/investigation. B.D.P., J.M., R.M., S.J.C.L., J.B.P., S.V., S.G.M., J.W.D., D.W.K., J.M.P., P.J.H., M.P.M., D.A.A., J.B.F. contributed to analysis/interpretation of data. B.D.P., J.M., J.B.F. contributed to manuscript writing. B.D.P., J.M., R.M., J.W.D., S.C.L., J.B.P., S.V., S.G.M., D.W.K., P.J.H., J.P., M.P.M., D.A.A., J.B.F. contributed to manuscript review/editing.

## Supplementary Material


