# Supplementary material for: Hospital Surgical Volume Is Poorly Correlated With Delivery of Multimodal Treatment for Localized Pancreatic Cancer: A National Retrospective Cohort Study
Source: Ann Surg Open. 2022 Aug 17;3(3):e197. doi: 10.1097/AS9.0000000000000197 (PMC9508964; doi:10.1097/AS9.0000000000000197)
Supplement: Supplementary file 1 [file as9-3-e197-s001.pdf]

Supplemental File 1: Adaption of Donabedian Model of Healthcare Quality

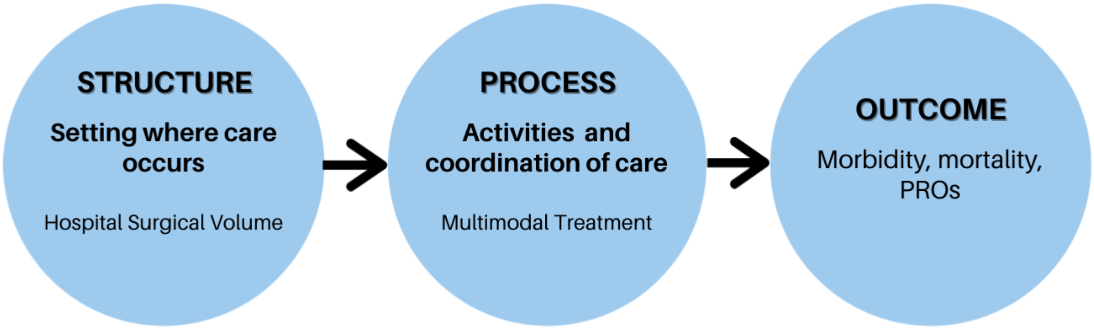

Supplemental File 2: Study population selection criteria

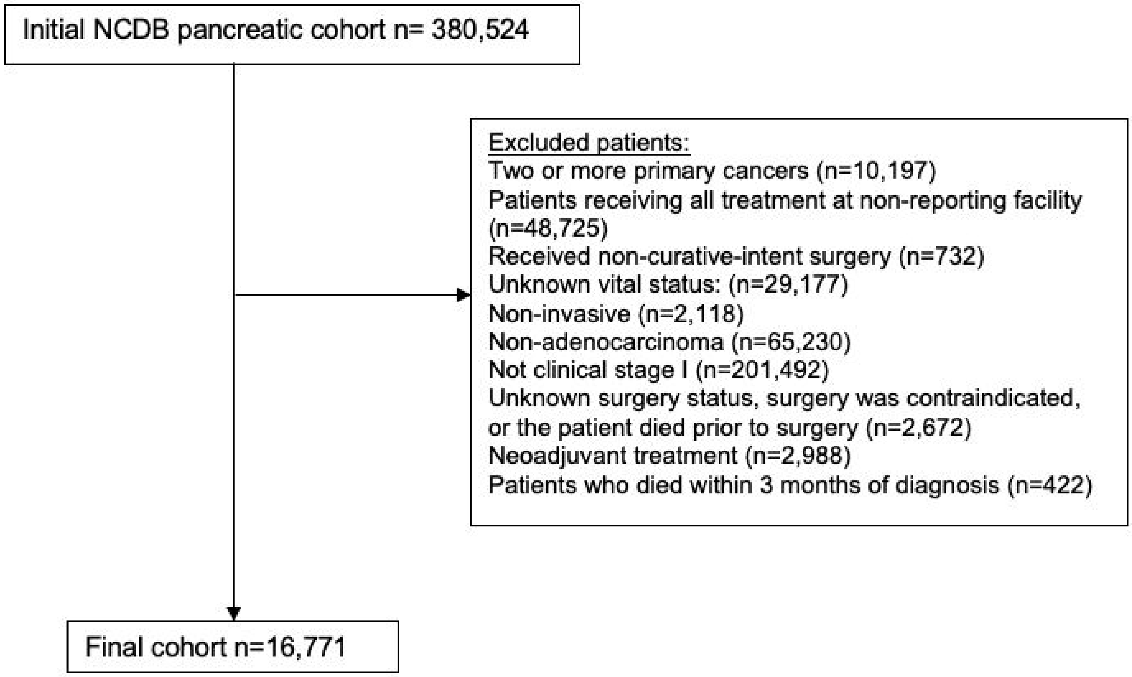

Supplemental File 3: Additional Characteristics of Resectable Pancreatic Cancer by Hospital Multimodal Treatment Delivery (n=16771)

	**Hospital Multimodal Treatment Delivery**			
	**Low (0-25%)**	**Moderate (>25-50%)**	**High (>50%)**	**p value**
Total	4763 (28.4)	8804 (52.5)	3204 (19.1)	
Charlson Comorbidity				***0.01***
0	3106 (65.2)	5554 (63.1)	1954 (61.0)	
1	1202 (25.2)	2370 (26.9)	920 (28.7)	
2	309 (6.5)	625 (7.1)	229 (7.1)	
3	146 (3.1)	255 (2.9)	101 (3.2)	
T stage				***<0.001***
T1	848(17.8%)	1671(19.0%)	600(18.7%)	
T2	2689(56.5%)	5251(59.6%)	1894(59.1%)	
T3	909(19.1%)	1628(18.5%)	610(19.0%)	
Unknown	317(6.7%)	254(2.9%)	100(3.1%)	
N Stage				***<0.001***
N0	923 (19.4%)	2552 (29.0%)	1052 (32.8%)	
N1	541 (11.4%)	2183 (24.8%)	1034 (32.3%)	
N2	301 (6.3%)	1303 (14.8%)	609 (19.0%)	
Unknown	2998 (62.9%)	2766 (31.4%)	509 (15.9%)	
Grade				***<0.001***
Well-differentiated	349 (7.3%)	728 (8.3%)	252 (7.9%)	
Moderately-differentiated	1164 (24.4%)	3247 (36.9%)	1523 (47.5%)	
Poorly-differentiated	776 (16.3%)	2318 (26.3%)	926 (28.9%)	
Unknown	2474 (51.9%)	2511 (28.5%)	503 (15.7%)	

Supplemental File 4: Relative Hazard of Death: Multivariable Cox Proportional Regression Full Model

	**HR (95% CI)**	**P-value**
Age	1.01 (1.01-1.02)	***<0.001***
Charlson Score	1.09(1.07-1.12)	***<0.001***
Race (ref: White)		***0.01***
Black	1.05 (0.99-1.11)	0.54
Unknown/Other	0.91 (0.84-1.00)	0.05
Insurance (ref: Uninsured)		***<0.0001***
Private	0.82 (0.72-0.93)	***0.003***
Medicaid	1.00 (0.86-1.16)	0.99
Medicare	0.87 (0.77-0.99)	***0.04***
Other government insurance	0.84 (0.69-1.02)	0.08
Unknown	0.96 (0.79-1.18)	0.75
Grade (ref: Well-differentiated)		***<0.001***
Moderately differentiated	1.31 (1.21-1.41)	***<0.001***
Poorly/undifferentiated	1.74 (1.61-1.88)	***<0.001***
Unknown	2.50 (2.32-2.70)	***<0.001***
Path T staging (ref: T1)		***<0.001***
T2	1.46 (1.39-1.53)	***<0.001***
T3	1.60 (1.51-1.70)	***<0.001***
Unknown	1.39 (1.27-1.53)	***<0.001***
Hospital surgical volume (ref: Lowest (0-25th percentile))		***<0.001***
Low (26-50th percentile)	0.96 (0.92-1.01)	0.16
High (51-75th percentile)	0.81 (0.77-0.85)	***<0.001***
Highest (76-100th percentile)	0.79 (0.75-0.83)	***<0.001***
Hospital multimodality treatment (ref: Low (0-25%))		***<0.001***
Moderate (>25-50%)	0.91 (0.87-0.95)	***<0.001***
High (>50%)	0.81 (0.77-0.86)	***<0.001***
